# Supplementary material for: Hebbian plasticity induced by temporally coincident BCI enhances post-stroke motor recovery
Source: Sci Rep. 2024 Aug 12;14:18700. doi: 10.1038/s41598-024-69037-8 (PMC11319604; doi:10.1038/s41598-024-69037-8)
Supplement: Supplementary file 1 — Supplementary Figure 1. [file 41598_2024_69037_MOESM1_ESM.pdf]

Hebbian plasticity induced by temporally coincident BCI enhances post-stroke motor recovery

Johanna Krueger[1], Richard Krauth[1], Christoph Reichert[2], Serafeim Perdikis[3], Susanne Vogt[1,4,5], Tessa Huchtemann[1,4,6], Stefan Dürschmid[2,7], Almut Sickert[8], Juliane Lamprecht[8,9], Almir Huremovic[8,10], Michael Görtler[4], Slawomir J. Nasuto[11], I-Chin Tsai[1], Robert T. Knight[12,13], Hermann Hinrichs[2,4,7], Hans-Jochen Heinze[2,14], Sabine Lindquist[15], Michael Sailer[8], Jose del R. Millán[16-19], Catherine M. Sweeney-Reed[1,-7*]

*Corresponding author: Catherine M. Sweeney-Reed, Neurocybernetics and Rehabilitation, Dept. of Neurology, Otto von Guericke University, Magdeburg, Leipziger Str. 44, 39120 Magdeburg, Germany. catherine.sweeney-reed@med.ovgu.de

Supplementary Figure 1

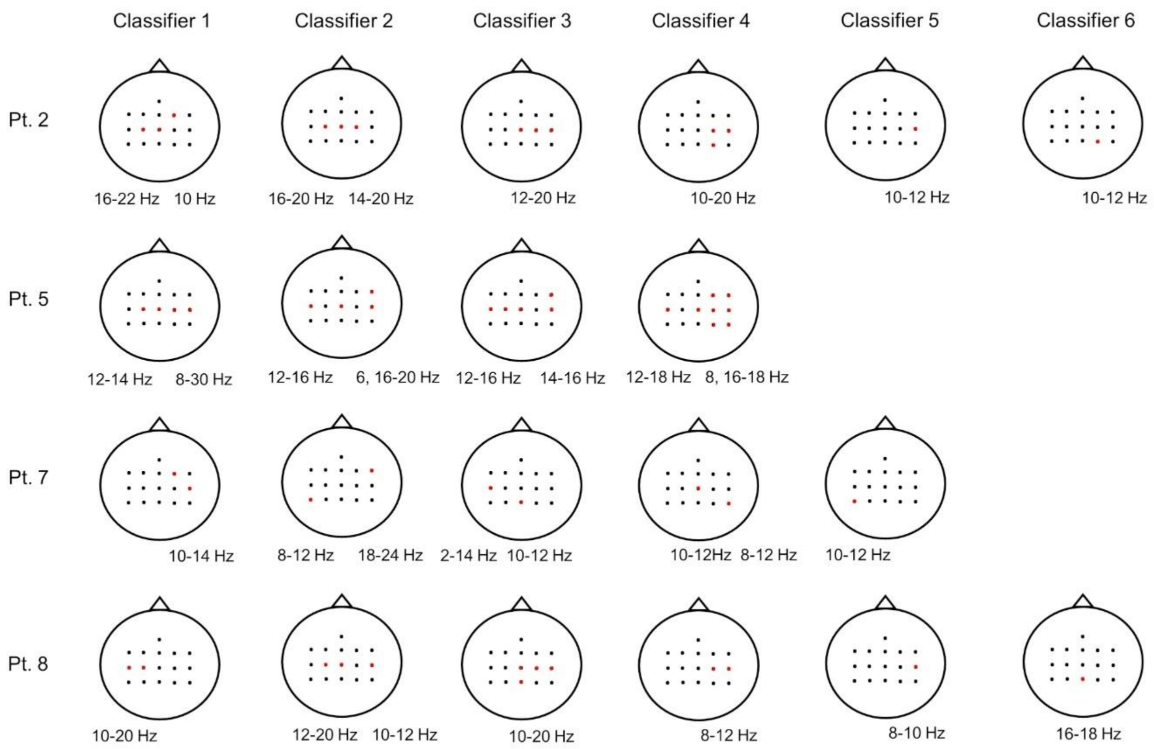

Supplementary Figure 1. **Classifier features.** Features selected for classifiers for patients in the BCI-FES group over the course of the therapy program. Note that classifiers were not trained to determine the timing of functional electrical stimulation in the Random-FES group. Selected electrodes are highlighted in red, and the frequencies chosen are given below the relevant electrode.
