# Supplementary material for: Hebbian plasticity induced by temporally coincident BCI enhances post-stroke motor recovery
Source: Sci Rep. 2024 Aug 12;14:18700. doi: 10.1038/s41598-024-69037-8 (PMC11319604; doi:10.1038/s41598-024-69037-8)
Supplement: Supplementary file 2 — Supplementary Information. [file 41598_2024_69037_MOESM2_ESM.pdf]

Hebbian plasticity induced by temporally coincident BCI enhances post-stroke motor recovery

Johanna Krueger[1], Richard Krauth[1], Christoph Reichert[2], Serafeim Perdikis[3], Susanne Vogt[1,4,5], Tessa Huchtemann[1,4,6], Stefan Dürschmid[2,7], Almut Sickert[8], Juliane Lamprecht[8,9], Almir Huremovic[8,10], Michael Görtler[4], Slawomir J. Nasuto[11], I-Chin Tsai[1], Robert T. Knight[12,13], Hermann Hinrichs[2,4,7], Hans-Jochen Heinze[2,14], Sabine Lindquist[15], Michael Sailer[8], Jose del R. Millán[16-19], Catherine M. Sweeney-Reed[1,-7*]

[*]Corresponding author: Catherine M. Sweeney-Reed, Neurocybernetics and Rehabilitation, Dept. of Neurology, Otto von Guericke University, Magdeburg, Leipziger Str. 44, 39120 Magdeburg, Germany. catherine.sweeney-reed@med.ovgu.de

Supplementary Information: Permutation tests

Clinical evaluation

Permutation testing showed a significant improvement in FMA-UE score over time in the BCI-FES group (p = 0.040, effect size = 1.27) but not in the Random-FES group (p = 0.39, effect size = 0.53). A significant improvement was also observed in the NIHSS score over time in the BCI-FES group (p = 0.045, effect size = 1.06) but not in the Random-FES group (p = 0.44, effect size = 0.53).

Applying permutation tests separately to the subgroup of patients who started therapy in the acute and subacute phase, the differences showed an analogous pattern to that observed on applying an ANOVA. The BCI-FES group starting treatment in the acute phase showed a trend toward a difference (p = 0.070, effect size = 1.40), which was absent in the patients starting in the subacute phase (p = 0.16, effect size = 0.98). The difference was not significant in the Random-FES group starting treatment in the acute (p = 0.41, effect size = 0.98) or in the subacute phase (p = 0.40, effect size = 0.56).

TMS

Applying permutation tests to the TMS measurements, the improvements were significant for both groups: BCI-FES group (p = 0.040, effect size = 1.24); Random-FES group (p = 0.048, effect size = 0.82), with a greater increase over time in the BCI-FES than the Random-FES group (p = 0.040, effect size = 1.17).

High-density EEG

Comparing upper beta (15-23 Hz) oscillatory power, 0.5-1.5 s after the movement cue, between pre- and post-treatment, permutation tests showed the difference was also significant in the BCI-FES (p = 0.010, effect size = 1.77) but not in the Random-FES group (p = 0.44, effect size = 0.52).

Permutation testing showed a trend towards a difference between LRTC pre- and post-treatment in the BCI-FES (p = 0.095, effect size = 1.02) but not the Random-FES (p = 0.76, effect size = 0.16) group. On an individual level, all BCI-FES group patients showed an overall mean reduction in beta LRTC, compared with only one patient in the Random-FES group (Figure 7C).

EEG–EMG coherence in the time–frequency window in which spectral power changed from pre- to post-treatment in the BCI-FES group (0.5-1.5 s; 15-23 Hz), at the electrode location over the contralateral primary motor cortex at which the power difference was greatest (C2), differed significantly between pre- and post-treatment on permutation testing in the BCI-FES (p = 0.045. effect size = 1.72) but not the Random-FES (p = 0.95, effect size = -0.059) group.
